# Development and optimization of cactus pear fruit jelly supplemented with *Moringa oleifera* leaf extract

**DOI:** 10.1016/j.heliyon.2022.e09587

**Published:** 2022-05-30

**Authors:** Kiros Mezgebo Akelom, Tadesse Yimer Bisetegn, Tizazu Yirga Bereka

**Affiliations:** a Department of Food Science and Postharvest Technology, College of Agriculture and Environmental Sciences, Adigrat University, Adigrat, Ethiopia; b Department of Agro-Processing Technology, Ethiopian Technical University, Addis Ababa, Ethiopia; c Department of Postharvest Management, College of Agriculture and Veterinary Medicine, Jimma University, Jimma, Ethiopia

**Keywords:** Nutritional composition, Sensory acceptability, D-optimal mixture design, Postharvest loss, Malnutrition

## Abstract

Cactus pear fruit and Moringa (*Moringa oleifera* Lam) are nutritionally abundant food sources. This study was conducted to evaluate the potential of cactus pear fruit for jelly development with the supplementation of *M. oleifera* leaves extract as means of postharvest loss, food insecurity and malnutrition reduction. D-optimal mixture design in Minitab Version 16 Statistical Software was used to generate ten experimental runs (formulations for jelly development) using 60–80% cactus fruit juice (CFJ), 0–20% *M. oleifera* extract (MOE), and 20–40% table sugar (TS). The developed jellies were analyzed for proximate composition, mineral content (Fe, Ca and Zn) and sensory evaluation. Nutritional and sensorial optimization was carried through a graphical approach using a D-optimal mixture design. The results indicated a significant difference in protein, fat, fibre, ash, carbohydrate, energy, iron, calcium, zinc, appearance, aroma, and taste amongst the formulated jellies (p < 0.05). In contrast, the significant difference was not observed in mouth feel and overall acceptability amongst the jellies. The overall optimum nutritional and sensorial attributes of the jelly were found in a range of CFJ (70–73%), MOE (3–14%) and TS (20–26%). However, developing jelly with the formulation of CFJ (68 %), MOE (12%) and TS (20%) was predicted to give the highest nutritional value and sensory acceptability score. The optimized result indicated the jelly would contain 3.97% protein, 0.92% fat, 1.09% fiber, 1.19% ash, 62.95% carbohydrate, 275.97 kcal/100 ​g energy, 98.45 mg/100 ​g calcium, 0.25 mg/100 ​g zinc, 7.43 mg/100 ​g iron and overall sensory acceptability score of 4.38 in five-point hedonic scale.

## 1 Introduction

Cactus pear (*Opuntia ficus-indica*) is the most widely known fruit of prickly pear, being characterized by a thin-skinned fruit with a juicy consistency and sweet pulp (Valdez-Cepeda et al., 2014; Cruz-bravo et al., 2019). It is native to Mexico that has spread worldwide and cultivated in marginal agricultural lands with low water availability in arid and semi-arid areas of different countries including Mexico, Argentina, Chile, Peru and Bolivia (America); Lebanon, Jordan, Israel and Syria (Middle East, Asia); Ethiopia, Algeria, South Africa, Tunisia and Morocco (Africa); Spain, Italy and Portugal (Europe), and in Australia (Oceania) (Valle-ortiz et al., 2019; Zegbe, 2020). Cactus pear fruit is highly appreciated worldwide for its active nutrients and multifunctional properties (Monroy-Gutierrez et al., 2017). It is a rich source of proteins, dietary fibres, minerals and phytochemicals such as betalains and β-carotene, lipid-soluble antioxidants and various phenolic compounds including quercetin, myricetin, kaempferol, luteolin, among others (Cruz-bravo et al., 2019).

In Ethiopia and other parts of the world, cactus pear fruit is mostly consumed fresh as a result, it is prone to postharvest loss due to fungal and bacterial proliferation that leads the fruit to decay and deteriorate rapidly (both in appearance and quality) within a few days at ambient storage conditions and limit its marketability (Valle-ortiz et al., 2019). In Ethiopia, the cactus pear fruits are produced in a tremendous amount in the Tigray region where it serves as a source of food and income particularly for the low-income householdes. Study showed that cactus pear fruit production covers 7.4% (379,338 ha) of the total land of the Tigray region (Gebretsadik et al., 2013). However, the quick spoilage nature of the fruits and limited value addition practices in the area causes substantial loss of this high-value food security crops and diminishes the farmers’ income. It has been identified that cactus pear fruit can be processed into jelly, jam and juice (Shumye Adilu et al., 2020) in order to reduce its postharvest loss and enhance its diversity for people usage. But, there was no sufficient scientific data and literatures in Ethiopia, particularly in the Tigray region that indicate the suitability and usage of cactus pear fruit in value addition practices.

On the other hand, food insecurity and malnutrition particularly chiled malnutrition is highly prevalent in Ethiopia including the Tigray region (Motbainor et al., 2015; Akombi et al., 2017) while the country is gifted with a nutritionally rich source of foods such as *Moringa oleifera (M. oleifera).* According to Ethiopian Central Statistical Agency (CSA) (2017) report, Ethiopian demographic and health survey findings showed that in 2016, 38% of children under five in Ethiopia were stunted (too short for their age), 10% were wasted (too thin for height) and 24% were underweight (too thin for their age). In addition, stunting (indication of chronic undernutrition) among children was greater in rural areas (41%) than in urban areas (26%) which range from a high of 49% in Tigray to a low of 14% in Addis Ababa (CSA, 2017). *M. oleifera* is utilized as a source of food and food products in tropical and subtropical regions of many African countries including Ghana, Nigeria, East Africa and Malawi (Oyeyinka et al., 2018) due to its significant nutritional, antioxidant and phytochemical benefits as well as its adaptability to survive in different climatic conditions (Falowo et al., 2018).

*M*. *oleifera* is a plant endowed with nutritionally important minerals (Ca, K, Zn, Mn, Fe and Cu), phytochemicals (phenolics, different enzymes, vitamins, alkaloids, tannins, steroids, terpenoids, flavonoids, saponins and anthraquinones) and proteins (essential amino acids such as methionine, tryptophan, lysine and cysteine) (Shousha et al., 2019). Every part of the plant (leaf, stem and root) can be used for nutritional or medicinal values due to its rich content of phytochemicals such as β-carotene and dietary antioxidants (Oyeyinka et al., 2018; Matic et al., 2018). Owing to its high nutritional and medicinal values, the incorporation of *M*. *oleifera* in various food product developments is an indispensable option to mitigate malnutrition and improve food security in regions where severe malnutrition and food insecurity are prevalent. This plant has been used in many African countries as food supplementation in amala (stiff dough), ogi (maize gruel), bread, biscuits, cheese, yoghurt and in making soups (Oyeyinka et al., 2018). However, to the knowledge of the researchers there was limited information about the utilization of *M*. *oleifera* extract as supplementation in jelly production.

Therefore, this study was aimed to investigate the potentials of cactus pear fruit for the development of nutrient-rich jelly supplemented with a *M*. *oleifera* leave extract. This understanding would help to devise a strategy to reduce the postharvest loss of cactus pear fruit and burdens of food insecurity and malnutrition in population as well as increase the utilization of various nutritionally rich agricultural produces including *M*. *oleifera*.

## 2 Materials and methods

### 2.1 Experimental materials

**Figure 1:**
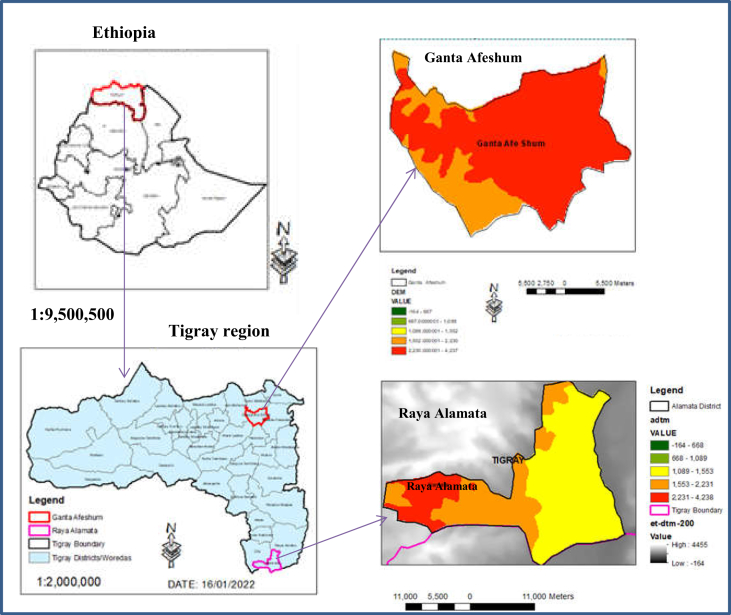
Sample collection areas (selected districts) in Tigray region, Ethiopia.

**Figure 2:**
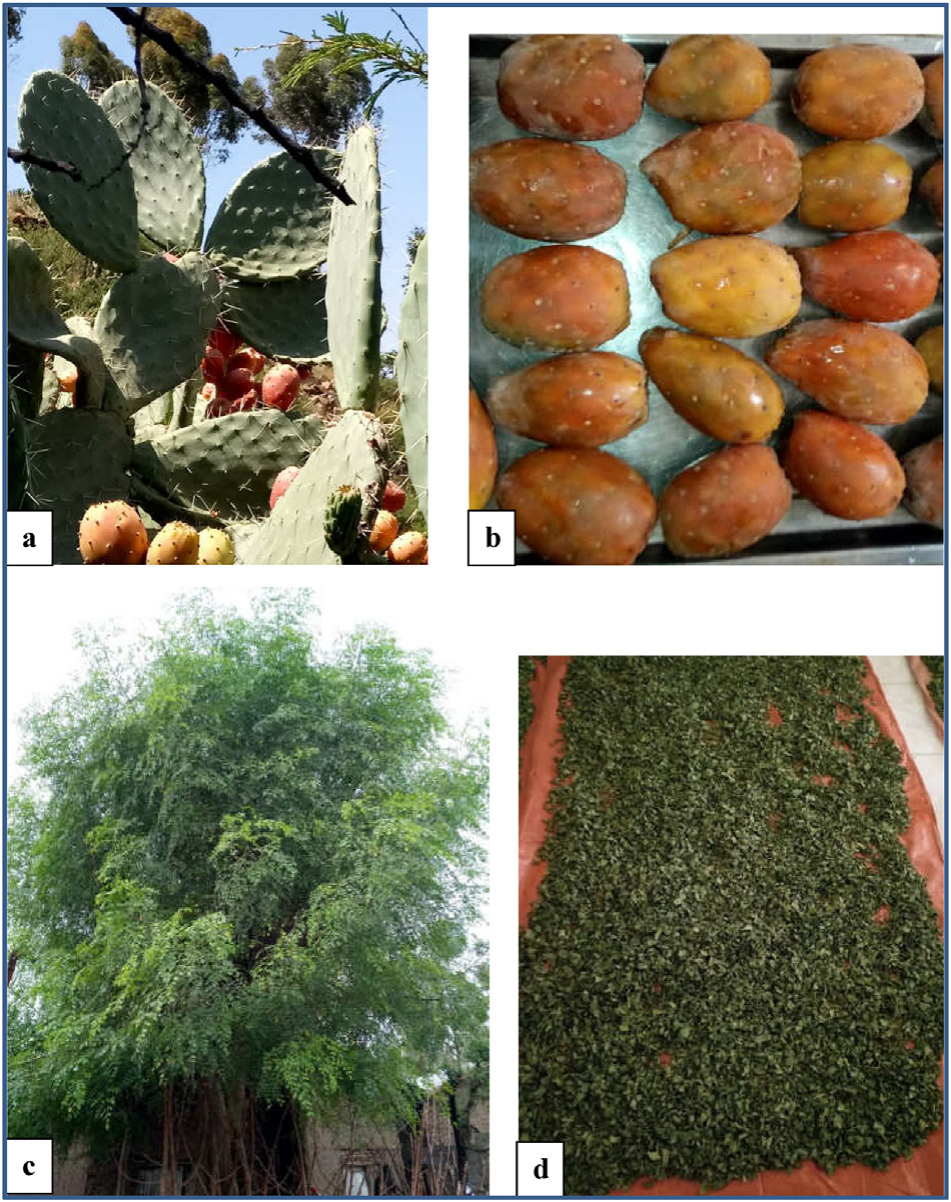
Plant material: a) cactus pear plant, b) cactus pear fruit, c) *M. oleifera* plant and d) dried *M. oleifera* leaves.

Matured (orange-yellow colour) cactus pear fruits (spiny (*Ashaque)* variety) (Figure 2a) and *M*. *oleifera* leaves (locally grown) (Figure 2c) were obtained from farmers in Ganta Afeshum district, Eastern Tigray and Raya Alamata District, Southern Tigray, Ethiopia respectively in July (Figure 1). Ganta Afeshum district is located about 921 Km far from Addis Ababa and 115 Km from Mekelle town (the capital city of Tigray regional state). Its annual average rainfall and temperature ranges from 140 mm to 672 mm and 6 °C to 30 °C, respectively (Tesfay et al., 2021). Raya Alamata is located about 600 km and 180 km far from Addis Ababa and Mekelle, respectively. Its annual average rainfall ranges from 299 mm to 1067 mm, with average monthly minimum and maximum temperatures of 14.8 °C and 26.97 °C, respectively (Eyasu et al., 2020). Table sugar (sucrose) was purchased from the local market in Adigrat, in Tigray regional state of Ethiopia.

### 2.2 Preparation of cactus peer fruit juice

**Figure 3:**
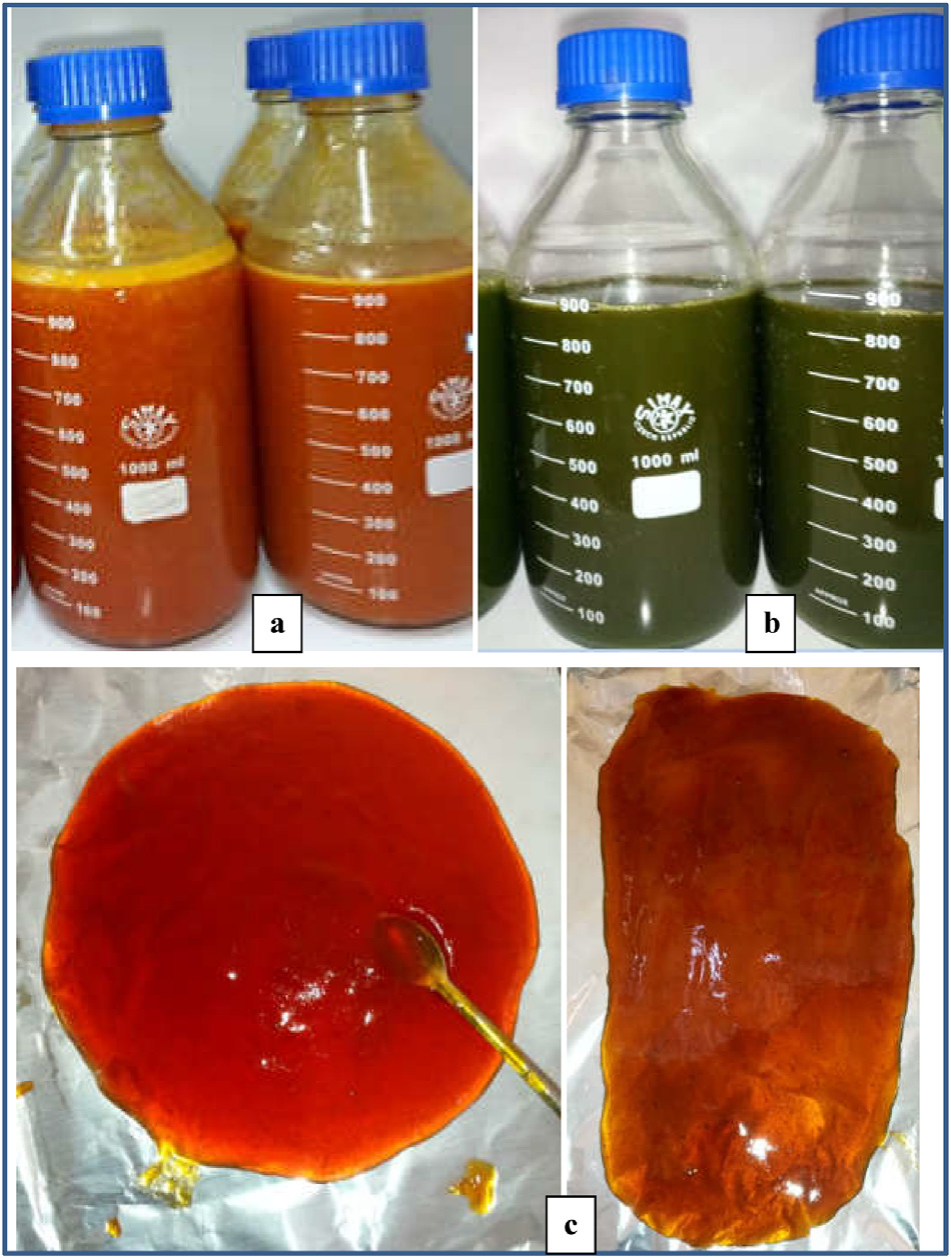
Products of cactus pear fruit and *M. oleifera*: a) cactus pear fruit juice, b) *M. oleifera* extracts and c) cactus pear fruit based jellies.

The cactus pear fruit juice was prepared following a procedures described by El-Samahy et al. (2006) with sligh modifications. Healthy cactus pear fruits (Figure 2b) were sorted, the skin part is scrubbed using a scrubbing pad to remove its thorn and washed using tap water to remove dirt particles. The fruits were cooled to 4 °C using a refrigerator at 95% relative humidity before peeling. Then the fruits were manually peeled and sliced into pieces using stainless steel knife and homogenized for 20 s in a blender (Moulinex, type 241, code 222, France) to ease the removal of the seeds from the flesh. The cactus fruit juice (Figure 3a) was separated from its seeds using a filter funnel and was stored in the refrigerator at 4 °C prior to jelly preparation.

### 2.3 Preparation of *Moringa oleifera* extract

*M. oleifera* extract (Figure 3b) was prepared following a procedure's described by Doerr and Cameron (2005) and Madukwe et al. (2013) with a slight modification. The *M. oleifera* stems with young and tender leaves were harvested. The leaves were stripped off from the stem, rinsed in clean water to remove the dirt and dried using atmospheric air in a shaded area to prevent the loss of vitamins (Vitamin A) by the impact of direct sunlight. The dried leaves (Figure 2d) were powdered using a blender and passed through a 0.5 mm sieve to obtain a uniform particle size. Then, fifty grams of the leaf powder was soaked for 30 min in 500 mL of hot water (Boiled water) and the resulting extract was drained off using a muslin cloth. The clear juice extract was pasteurized at 62 °C for 30 min and stored in the refrigerator at 4 °C prior to usage for jelly preparation.

### 2.4 Experimental design

Simplex Centroid Design was used to generate ten experimental units from three ingredients (Cactus fruit juice (CFJ), *M.* oleifera Extract (MOE) and Table Sugar (TS)) using D-optimal Mixture design in Minitab version 16 statistical software. The lower and upper percentage values of the three ingredients (CFJ: 60%–80%, MOE: 0%–20%, and TS: 20%–40%) were determined considering previous research findings on jelly preparation (Fernandes et al., 2015; Curi et al., 2017; Purba et al., 2018; Panchal et al., 2018).

### 2.5 Preparation of cactus fruit based jelly

Ten blends of cactus fruit juice (Figure 3a), *M. oleifera* leaf extract (Figure 3b) and sugar were prepared as per the generated ten experimental combinations. The jellies (Figure 3c) were prepared following the method of Panchal et al. (2018) with slight modification. 5% pectin and 10% of lemon juice were added in each treatment combination while the blends were boiling in the cooking pan at 100 °C (continuous stirring was employed during boiling to avoid coagulation and sticking on the cooking pan). The contents were allowed to continue boiling until it formed a jell and attained the required degree of consistency (until its total soluble solid reached a °Brix of 67) (Islam et al., 2012; Panchal et al., 2018). The jelly was poured into a sterilized glass bottle, cooled down to room temperature and stored in the refrigerator at 4 °C until further analysis.

### 2.6 Proximate composition

Moisture (MC) (hot air oven method), crude protein (Kjeldahl method), crude fat (Soxhlet extraction method), crude fibre (non-enzymatic gravimetric method) and ash contents of the samples were determined by official method with method numbers of 925.10, 979.09, 2003.06, 920.168 and 923.03, respectively (Horwitz, 2010). The total carbohydrate content and total gross energy value were obtained using the difference method (Onyeike et al., 1995) and the method developed by Osborne & Voogt (1978), respectively.

### 2.7 Mineral analyses

**Figure 4:**
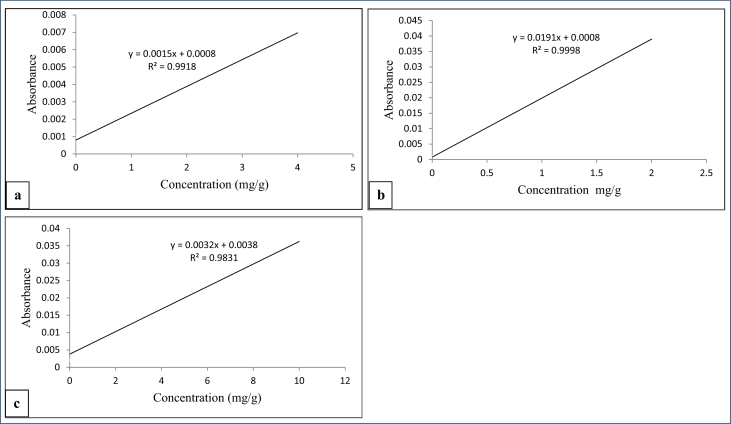
Mineral standard calibration curves: a) iron, b) zinc and c) calcium.

The content of Ca, Zn and Fe in jellies were measured by atomic absorption spectrophotometer (PerkinElmer, Model 3100, USA) (Hernández et al., 2005). Thus, 5 ​g of sample was dry ashed and dissolved in 3 mL of concentrated nitric acid after weighing. Then the dissolved sample was diluted with distilled water in a 25 mL calibrated flask and the resulting solution was used to determine the concentration of Ca, Zn, and Fe. Ca, Z and Fe standard stock solutions were prepared with a proper dilution of stranded pure metals. Air-acetylene has been used as a source of energy for the atomization of the samples and standards (AACC 2000). To determine the level of iron, zinc and calcium in the sample, absorbance was measured at 248.3 nm, 213.8 nm and 422.7 nm respectively. The level of concentration was estimated respectively using the standard calibration curve prepared from analytical grades of iron wire (Figure 4a), ZnO (Figure 4b) and Ca (Figure 4c).

### 2.8 Sensory evaluation

The formulated jellies were subjected to a five-point hedonic scale (where 1 = dislike very much, 2 = dislike slightly, 3 = neither like nor dislike, 4 = like slightly, and 5 = like very much (Lim, 2011)) sensory analysis. 50 untrained panellists were randomly selected, briefly introduced about five-point hedonic scale measurements and asked to assess the appearance, aroma, taste, mouthfeel and overall acceptability of the samples. Samples were freshly prepared and served in white plates which were coded randomly. During the evaluation, panellists were instructed to palate clean with water between each sample tasting.

### 2.9 Ethical consideration

This study was approved by the Institutional Review Board of Adigrat University and conducted as per the established research ethical guideline of the university. For the sensory evaluation part informed consent was obtained from each of the participants prior to the evaluation.

### 2.10 Data analysis

Minitab statistical software (version 16) was used to analyse the data. Analysis of variance (ANOVA) was used to determine the statistical significance of the terms in the regression equations at a 5% significance level (p < 0.05). The normal distribution of the data was checked and the fitted models were generated for all parameters. To determine the optimum formulation of CFJ based jelly with substitution of different levels of MOE and TS, graphical optimization was carried out considering the best nutrient composition and sensory characteristics of the jelly (Montgomery et al., 2012).

## 3 Results and discussions

### 3.1 Proximate compositions of the cactus pear fruit-based jellies

The proximate compositions of the cactus pear based jellies and their respective p-values are indicated in Table 1 and Table 2, respectively. Significant differences were observed amongst the moisture contents of all the jellies in the different levels of interactions (p < 0.05) (Table 2). The highest MC value (31.61%) was obtained from jelly prepared from 80% CFJ, 0% MOE and 20% TS (FM jelly 1 in Table 1), while the lowest MC value (27.50%) from 60% CFJ, 0% MOE and 40% TS prepared jelly (FM jelly 5 in Table 1). The result indicated that the MC of the jellies was decreased with the increasing substitution of TS in the formulation from 20% to 40%. This was in agreement with Ahmed et al. (2016) who reported the decreased levels of MC in the sapodilla fruit (*Manilkara zapota*) jam as the sugar ratio was increasing. The MC determines the duration of the food that can be stored without significant deterioration (Fellows, 2009) and increased levels of sugar in food usually decreases the amount of water available to support the growth of microorganisms (sugar withdraws water from microorganisms and retardes their growth), thus, in turn, improving the shelf life of the food (Amit et al., 2017).

**Table 1:** Proximate composition, energy and mineral content of different cactus fruit based jellies.

Formulation	Components (%)			Proximate Composition (%)						Energy	Minerals (mg/100 ​g)		
	CFJ	MOE	TS	MC	Ash	CP	CFA	CFI	CHO	(kcal/100 ​g)	Ca	Zn	Fe
FM Jelly 1	80.0	0.0	20.0	31.61	0.70	0.78	0.33	0.25	66.33	271.43	16.66	0.02	0.23
FM Jelly 2	73.3	3.3	23.3	29.54	0.88	1.35	0.43	0.41	67.40	278.88	21.98	0.07	2.45
FM Jelly 3	70.0	10.0	20.0	30.00	1.15	3.25	0.89	1.00	63.71	275.81	73.66	0.20	6.74
FM Jelly 4	63.3	3.3	33.3	27.75	0.73	1.10	0.40	0.32	69.70	286.82	16.92	0.05	2.14
FM Jelly 5	60.0	0.0	40.0	27.50	0.16	0.10	0.15	0.02	72.07	290.04	10.01	0.01	0.14
FM Jelly 6	60.0	20.0	20.0	29.89	1.25	7.56	0.99	1.35	58.96	274.98	229.77	0.45	8.83
FM Jelly 7	63.3	13.3	23.3	29.24	1.23	4.35	0.91	1.09	63.17	278.24	109.84	0.27	7.60
FM Jelly 8	70.0	0.0	30.0	28.10	0.63	0.74	0.16	0.02	70.35	285.76	10.30	0.01	0.19
FM Jelly 9	66.7	6.7	26.7	28.20	1.04	2.12	0.61	0.66	67.38	283.46	37.81	0.12	4.72
FM Jelly 10	60.0	10.0	30.0	27.99	1.10	2.93	0.80	0.89	66.31	284.08	64.18	0.19	6.43

CFJ = Cactus fruit juice; MOE = *Moringa oleifera* extract; TS = Table sugar.

Where: FM = Formulation, CFJ = Cactus fruit juice, MOE = Moringaoleiferaextract, TS = Table sugar, MC = Moisture content, CFA = Crude fat, CFI = Crude fiber, CP = Crude protein, CHO = Carbohydrate, Kcal = Kilo Calorie, Ca= Calcium, Zn = Zinc and Fe=Iron.

**Table 2:** Analysis of variance (ANOVA), p-values of proximate composition, mineral contents (Fe, Ca, Zn) and sensory properties of cactus fruit-based jellies.

Sources	Proximate composition							Minerals			Sensory analysis				
	MC	Ash	CP	CFA	CFI	CHO	En	Ca	Zn	Fe	App	Aroma	Taste	MF	OA
Linear	0.01	0.00	0.00	0.01	0.01	0.00	0.03	0.00	0.00	0.01	0.02	0.03	0.00	0.12	0.15
Quadratic	0.01	0.00	0.00	0.01	0.00	0.01	0.01	0.00	0.00	0.00	0.01	0.04	0.00	0.74	0.40
A∗B	0.02	0.00	0.00	0.00	0.00	0.01	0.03	0.00	0.00	0.00	0.04	0.06	0.00	0.65	0.14
A∗C	0.00	0.00	0.00	0.04	0.01	0.01	0.00	0.01	0.04	0.54	0.07	0.06	0.01	0.37	0.69
B∗C	0.03	0.00	0.00	0.00	0.00	0.02	0.12	0.00	0.04	0.00	0.01	0.05	0.00	0.84	0.64

Where: MC = Moisture content (%), CP = Crude protein (%), CFA = Crude fat (%), CFI = Crude fiber (%), CHO = Carbohydrate (%), En = Energy (Kcal/100gm), Ca= Calcium (mg/100 g), Zn = Zinc (mg/100 g) and Fe=Iron (mg/100 g), App = Appearance, MF = Mouth feel, OA = Overall acceptability, A = Cactus fruit juice, B= Moringaoleiferaextract, and C=Table sugar.

The highest ash content (1.25%) was recorded in the jelly prepared from 60% CFJ, 20% MOE and 20% TS (FM jelly 6 in Table 1) while the lowest ash content (0.16%) corresponded to the jelly containing 60% CFJ, 0% MOE and 40% TS (FM jelly 5 in Table 1). A high significant difference (p < 0.05) was observed in ash contents of the jellies (between CFJ with MOE, CFJ with TS, MOE and TS) in linear and quadratic models. This finding observed the increasing levels of ash content with the incremental proportion of *M. oleifera* extract which is in line with the study of Manaois et al. (2013) who reported the increased levels of ash content with increasing *M. oleifera* substitution in the formulations. Shiriki et al. (2015) also reported a significant level of ash content increment in complementary food formulated from maize, soybean and peanut with supplementation of *M. oleifera* leaf powder. While Moyo et al. (2011) and Olusanya et al. (2020) reported the ash content of *M. oleifera* as 9.53% and 13.08% in the dry leaf and powder, respectively.

The protein conetent of the cactus pear based jellies was recorded in the range of 0.104%–7.56% (Table 1). Significant differences (p < 0.05) in the protein content were observed in the linear and quadratic model amongst the jellies of CFJ with MOE, CFJ with TS, and MOE with TS (Table 2). High protein content was observed in the jellies with the increasing level of *M. oleifera* extracts in the formulation. Similarly, Ajibola et al. (2015) and Sengev et al. (2013) reported the enhancement of the protein content in bread and biscuits supplemented with *M. oleifera* powders, respectively. Studies indicated that the dried leaves of *M. oleifera* contain an appreciable amount of protein (30.3% crude protein) and are reported as an important crop for the mitigation of malnutrition (Thurber and Fahey (2009); Moyo et al. (2011)). Shiriki et al. (2015) also reported the increased level of protein content in different foods supplemented with *M. oleifera* leaf powders. This finding confirmed that *M. oleifera* is a potential crop that could be used during formulation to enhance the protein content of various food items.

High significance differences (p < 0.05) were observed in crude fat contents of the jellies amongst the linear and quadratic model as well as in the interaction of CFJ with MOE, CFJ with TS and MOE with TS (Table 2). The highest fat (0.99%) content was obtained in the jellies made from 60% CFJ, 20% MOE, and 20% TS (FM jelly 6 in Table 1) while the lowest fat content (0.15%) was observed in 60% CFJ, 0% MOE, and 40% TS formulated jellies (FM jelly 5 in Table 1). The observed small change in the crude fat of the formulated jellies might be attributed to the moderate fat content of *M. oleifera* leaves (Owusu et al., 2008; Ogbe and Affiku 2021; Odinakachukwu et al., 2014). Studies reported that *M. oleifera* contains 6.5% and 8.38% fat content in its dry leaf (Moyo et al., 2011) and powder (Olusanya et al., 2020) respectively.

The crude fibre content of the formulated jellies was ranged from 0.02% to 1.35% (Table 1). High significant differences (P < 0.05) were observed in the crude fibre contents of the formulated jellies in the linear and quadratic models as well as in the interaction of CFJ with MOE, CFJ with TS, and MOE with TS (Table 2). This finding indicated that the crude fibre content of the jellies was increased with the increasing proportion of MOE supplementation. Shiriki et al., (2015) reported the increased level of fibre in the diet which is supplemented with .*M. oleifera* leaves powder. The increased level of crude fibre content in the formulated jellies may be attributed to the presence of the appreciable amount of crude fibre in *M. oleifera* leaves. Another study also reported that *M. oleifera* leaf is rich in crude fibre (19.24%) which play a key role in promoting human health (Odinakachukwu et al., 2014).

The carbohydrate content of the formulated jellies was ranged from 58.96%-72.07% (Table 1). High significant differences were observed (P < 0.05) in carbohydrate content of jellies in linear and quadratic models and in the interaction of CFJ with MOE, CFJ with TS and MOE with TS (Table 2). The carbohydrate content of the jelly was increased with the increasing proportion of TS in the formulations because TS is one of the carbohydrate components (Naeem et al., 2017). In contrast, a decline in carbohydrate content in the formulated jellies was observed with the increasing amount of MOE, this could be due to the lower carbohydrate content of MOE (Shiriki et al., 2015). According to Madukwe et al. (2013), *M. oleifera* leaf extract contains carbohydrate content of 2.63%.

The gross energy content of the formulated jellies was varied from 271.43 kcal/100 g - 290.04 kcal/100 g (Table 1). A high significant difference (P < 0.05) was observed in the gross energy of jellies in the linear and quadratic models and in the interaction of MOE with TS, CFJ with MOE, and CFJ with TS (Table 2). The energy content of the formulated jellies was increased with the increasing proportion of TS in the formulation; this may be due to the high energy content of the TS (4 kcal/g or ≈16 kcal/teaspoon) (Fitch and Keim, 2012).

### 3.2 Mineral contents of the cactus pear fruit-based jellies

The selected mineral content of the cactus pear based jellies and their respective p-values are indicated in Table 1 and Table 2 respectively. The highest calcium content (229.77 mg/100 ​g) was found in jelly prepared from 60% CFJ, 20% MOE, and 20% TS (FM jelly 6 in Table 1) while the lowest calcium content (10.01 mg 100 g-1) in jelly was obtained from 60% CFJ, 0% MOE and 40% TS (FM jelly 5 in Table 1). The calcium content of the formulated jellies showed a highly significant difference in the linear and quadratic models as well as in the interaction of CFJ with MOE, CFJ with TS, and MOE with TS (Table 2).

The highest zinc content (0.45 mg/100 g) was found in jelly prepared from 60% CFJ, 20% MOE and 20% TS (FM jelly 6 in Table 1), while the lowest content (0.01 mg/100 g) was found in jelly prepared from 60% CFJ, 0% MOE and 40% TS (FM jelly 5 in Table 1). The composition of zinc in the jellies showed a high significance difference (P < 0.05) both in the linear and quadratic models as well as in the interaction of CFJ with MOE, CFJ with TS and MOE with TS (Table 2).

The highest Fe content (8.83 mg/100 g) was found in the jelly prepared from 60% CFJ, 20% MOE, and 20% TS (FM jelly 6 in Table), whereas the lowest Fe content (0.14 mg 100 g-1) was found in jelly from 60% CFJ, 0% MOE, and 40% TS (FM jelly 5 in Table 1). Iron content was found to be highly significant (P < 0.05) in the jellies in the linear and quadratic models as well as in the interaction of CFJ with MOE, MOE with TS and CFJ with TS (Table 2).

This finding revealed that calcium, zinc and iron content were increased in appreciable amounts in all formulated jellies with the increasing proportion of *M. oleifera* in the formulation. Glover-Amengor et al., (2017) reported *M. oleifera* as a superior source in minerals content. Another finding also indicated *M. oleifera* leaves rich in calcium (1.34 ± 0.10 mg/100 g), zinc (7.49 ± 0.02 mg/100 ​g), and iron (26.44 ± 0.04 mg/100 g) contents (Odinakachukwu et al., 2014). While *M. oleifera* leaves extract reported to contain 2.07 ± 0.5 mg/100 g iron and 33.5 ± 0.92 mg/100 g calcium (Madukwe et al., 2013). On the other hand, cactus pear fruit juice prepared from pure pulp (without addition of water) was reported to contain 564 μg/g of Ca, 7.32 μg/g of Zn and 4.50 μg/g of Fe (Aregahegn et al., 2013).

### 3.3 Sensory evaluation of the cactus pear fruit-based jellies

Sensory evaluation results and their respective p-values of the cactus pear based formulated jellies are indicated in Table 3 and Table 2, respectively. A high significant difference (P < 0.05) was observed amongst the appearance evaluation results of the jellies in the linear and quadratic models as well as the interaction of MOE with TS and CFJ with MOE (Table 2). A significant difference was observed in aroma evaluation in the quadratic model and in the interaction of MOE with TS while for taste significant difference was observed in all cases (linear and quadratic models as well in all possible interactions). However, no significant difference was observed in all treatments for the case of mouthfeel (P < 0.05) and a significant difference was observed in the overall acceptability of the jellies in all treatments (Table 2).

**Table 3:** Sensory attributes of cactus pear fruit-based formulated jellies.

Formulation	Components (%)			Sensory Attributes (5 point hedonic scale)				
	CFJ	MOE	TS	Appearance	Aroma	Taste	Mouth Feel	OA
FM Jelly 1	80.0	0.00	20.0	4.84	4.95	4.85	4.90	4.88
FM Jelly 2	73.3	3.3	23.3	4.68	4.76	4.74	4.78	4.61
FM Jelly 3	70.0	10.0	20.0	4.61	4.66	4.63	4.48	4.44
FM Jelly 4	63.3	3.3	33.3	4.23	4.08	4.21	4.17	4.16
FM Jelly 5	60.0	0.00	40.0	4.14	3.86	4.13	4.11	4.10
FM Jelly 6	60.0	20.0	20.0	4.01	3.71	4.02	3.99	3.48
FM Jelly 7	63.3	13.3	23.3	4.19	4.20	4.24	4.26	4.25
FM Jelly 8	70.0	0.00	30.0	4.65	4.76	4.64	4.61	4.53
FM Jelly 9	66.7	6.7	26.7	4.27	4.36	4.38	4.33	4.29
FM Jelly 10	60.0	10.0	30.0	3.79	3.26	3.89	4.05	3.60

CFJ = Cactus fruit juice; MOE = *Moringa oleifera* extract; TS = Table sugar; OA = Overall acceptability; Values indicating that 1 = dislike very much, 2 = dislike slightly, 3 = neither like nor dislike, 4 = like slightly, and 5 = like very much.

Relatively high rankings were given for appearance, aroma, taste, mouthfeel and overall acceptability of jellies prepared from CFJ (80%), MOE (0%) and TS (20%) (FM jelly 1 in Table 1). In contrast, jellies prepared from CFJ (60%), MOE (10%) and TS (30%) (FM jelly 10 in Table 1) had less scores for appearance, aroma, taste, and overall acceptability. The study revealed that the sensory acceptability of the jellies was increased with decreasing the proportion of MOE in the formulation.

The decreased sensory acceptability of the jellies with increasing the proportion of MOE in the formulation maybe associated with the oxidation of the green color (chlorophyii) of *M. olifera* into brown color (pheophytin) during its drying process that result darker color when its promotion increased in the formulation and in return distract consumer interests (Priyanto and Nisa, 2016; Wulandari et al., 2020). The decreased sensory acceptability of the jelly with increasing of MOE in the formulation may also be associated with the production of unpleasant (distinctive) aroma from *M. olifera* leaves oil by lipoxidase enzyme and bitter taste of MOE due to its tannin content (Ardhanareswari, 2019). Soares et al. (2020) reported that bitterness in food is linked to tannin and produces a bitter taste in the mouth during consumption. Similar findings also reported that the addition of *M. oleifera* in different food samples resulted in a decrease in sensory acceptability (Boateng et al., 2019). Jellies prepared from a high proportion of CFJ and TS with moderate amount of MOE (13.3 %) supplementation were scored high consumer acceptability. In general, all the formulated jellies enhanced in all aspects of the sensory properties except the mouthfeel in the jelly prepared from CFJ (60%), MOE (20%) and TS (20%) (FM jelly 6 in Table 1).

### 3.4 Mixture optimization: overall nutritional and sensorial properties of jellies

**Figure 5:**
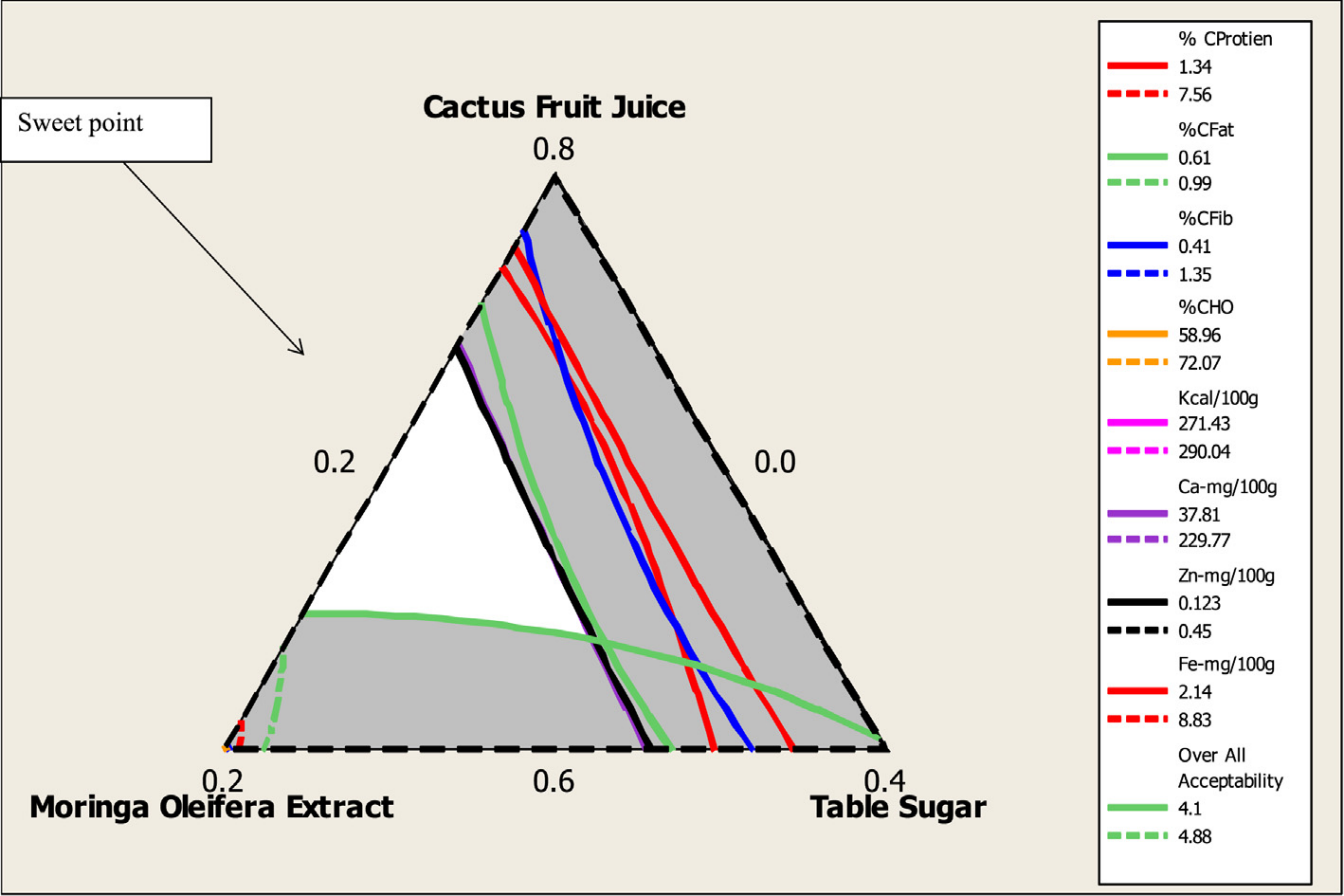
Overlaid contour plot of proximate, mineral and sensory quality of cactus fruit-based jellies.

Optimization helps to generate the best formulation with optimal proportions of ingredients for developing a food product with improved nutritional and sensorial properties (Prinyawiwatkul et al., 1997). The regions of acceptability in the contour plot were superimposed for protein, fat, carbohydrate, fibre, energy, minerals (Fe, Ca and Zn) and overall sensorial attributes to determine the optimal jelly formulation. The interest superimposed region of the contour plot (%crude protein, %carbohydrate, %crude fat, %crude fibre, energy kcal/100 ​g, (Fe, Zn and Ca) mg/100 ​g and overall acceptance; hedonic ratings) resulted in an optimum region for the jelly (Figure 5). The white region in this figure indicates that any point within this region represents an optimum combination of CFJ, MOE and TS, which results in an optimal level of nutritional and sensory attributes in the jelly. Thus, the overall optimum values were found in a range of 70–73% CFJ, 3–14% MOE and 20–26% TS (Figure 5). Within this combination, the jelly will contain 1.34%–7.56% protein, 0.61%–0.99% fat, 0.41%–1.35% fibre, 58.96%–72.07% carbohydrate, 271.43–290.04 kcal/100 ​g energy, 37.81–229.77 mg/100 ​g of Ca, 0.12–0.45 mg/100 ​g of Zn, 2.14–8.83 mg/100 ​g of Fe and 4.1–4.88 overall acceptability score in 5 point-hedonic scales.

Based on the lower and upper values of all the parameters used in this study, the best optimal treatment combinations for the jelly were found within the ratio of 68% CFJ, 12% MOE and 20% TS. This optimal treatment combinations was predicated to give 3.97% protein, 0.92% fat, 1.09% fiber, 1.19% ash, 62.95% carbohydrate, 275.97 kcal/100 ​g energy, 98.45 mg/100 ​g calcium, 0.25 mg/100 ​g zinc, 7.43 mg/100 ​g iron and overall sensory acceptability score of 4.38 in five-point hedonic scale (Table 4). Mezgebo et al., (2018) reported that overall optimization using mixture design improved the nutritional quality and sensory properties of porridge developed from the mixture of red teff flour, malted soybean flour and papaya fruit powder. Optimization studies of fruit juice using mixture design also reported to give a highly acceptable fruit punch with formulation of orange (33%), mango (66%) and lemmon (1%) (Kumar et al., 2010). In addition, Schiassi et al. (2019) obtained desirable nutritional and sensory properties of berry based jully using mixture design with recommended ideal mixtures of black berry (55%–100%), blue berry (0%–20%) and strawberry (0%–40%). Best fitted regression models and respective R^2^ values for nutritional, mineral and sensory acceptability of jelly are presented in Table 5.

**Table 4:** The predicated proximate, mineral and sensory qualities of cactus fruit based jelly for the overall optimized best treatment combination (68% CFJ, 12% MOE and 20% TS).

Proximate	Predicted Value	Minerals	Predicted Value	Sensory acceptability	Predicted Value (5point)
MC (%)	29.87	Ca mg/100g	98.45	Appearances	4.50
Ash (%)	1.19	Zn mg/100g	0.25	Aroma	4.59
Protein (%)	3.97	Fe mg/100g	7.43	Taste	4.54
Fat (%)	0.92			Mouth feel	4.41
Fiber (%)	1.09			Over all	4.38
CHO (%)	62.95				
Kcal/100g	275.97

**Table 5:** Regression models for the nutritional compositions, mineral (Fe, Zn, Ca) contents and sensory acceptability of cactus pear fruit-based jelly.

Properties	Regression model	R^2^ value
MC	47.3A + 68.2B + 85.7C-74.7AB-146.4AC -67.4BC	99.12%
Ash	-0.36A-11.31B-10.91C + 16.43AB+19.77AC+39.4BC	99.87%
Protein	-0.83A + 113.93B-15.34C-94.7AB+28.12AC-91.89BC	99.99%
Fat	1.33A-13.52B + 4.67C + 19.86AB-10.46AC+20.89	99.60%
Crude fiber	1.47A-10.4B + 5.37C + 18.5AB-12.54AC+19.06BC	99.90%
Carbohydrate	51.05A-46.9B + 30.53C + 114.64AB+121.49AC+80.23BC	99.87%
Calorie	212.9A + 146.4B + 102.8C + 258.5AB+504.3AC+141.4BC	99.41%
Calcium	45A + 5133B + 122C-4946AB-274AC-5556BC	100.00%
Zinc	0.095A + 4.83B + 0.298C-3.15AB-0.702AC+176.6BC	99.99%
Iron	2A-117.3B + 9.33C + 205.2AB-20.1AC+176.6	99.71%
Appearance	4.41A-1.13B-4.53C + 16.98AB+13.84AC-29.6BC	99.00%
Aroma	2.76A-10.4B-18.43C + 40.13AB+39.86AC-43.32BC	97.20%
Taste	4.45A-4.92B-4.86C + 19.81AB+14.17AC-18.96BC	99.69%
Mouth feel	5.053A + 0.034B-2.769C + 4.351AB+8.987AC-1.922BC	97.13%
Overall acceptability	4.94A-18.46B-2.07C + 34.07AB+8.09AC-9.24BC	93.38%

A = Cactus fruit juice, B= *Moringa oleifera* extract, and C= Table sugar.

## 4 Conclusions

In this study, jellies were developed from cactus pear fruit with the supplementation of *M. oleifera* leaves extract. The results indicated improvement of the contents of ash, protein, fat, fibre and minerals in the jelly with the increasing proportion of MOE (0–20%) in the formulation. While the carbohydrate and energy ratio of the product increased significantly with the increasing proportion of CFJ and TS. However, excessive amount of MOE in the formulation lowered sensory acceptability of the jelly. The 60% CFJ, 20% MOE and 60% TS resulted in a significant improvement in jelly nutritional qualities while 80% CFJ, 0% MOE and 40% TS resulted in higher sensory acceptability. Overall optimization of the ingredients (68% CFJ, 12% MOE and 20% TS) in the formulation indicated improvement in protein, fat, fibre, carbohydrate, energy, Ca, Zn, and Fe contents as well as overall sensorial acceptability. Thus, this finding suggested that processing can reduce postharvest loss of fruits, enhance their nutritional value with supplementation of nutritionally rich food items such as *M. oleifera* and their product can help to improve household food security and support the mitigation of malnutrition in the population. In addition, this study suggested further investigation in exploring the potential of mixture optimization for improving the phytochemical, fatty acid and amino acid profiles of cactus pear fruit based jelly.

## Declarations

### Author contribution statement

Kiros Mezgebo Akelom; Tadesse Yimer Bisetegn; Tizazu Yirga Bereka: Conceived and designed the experiments; Performed the experiments; Analyzed and interpreted the data; Contributed reagents, materials, analysis tools or data; Wrote the paper.

### Funding statement

Kiros Mezgebo Akelom was supported by 10.13039/501100022050Adigrat University [AGU/BI/001/2010].

### Data availability statement

Data included in article/supp. material/referenced in article.

### Declaration of interest’s statement

The authors declare no conflict of interest.

### Additional information

No additional information is available for this paper.
